# Development, scoring, and reliability of the Microscale Audit of Pedestrian Streetscapes (MAPS)

**DOI:** 10.1186/1471-2458-13-403

**Published:** 2013-04-27

**Authors:** Rachel A Millstein, Kelli L Cain, James F Sallis, Terry L Conway, Carrie Geremia, Lawrence D Frank, Jim Chapman, Delfien Van Dyck, Lindsay R Dipzinski, Jacqueline Kerr, Karen Glanz, Brian E Saelens

**Affiliations:** 1 SDSU/UCSD Joint Doctoral Program in Clinical Psychology, 3900 Fifth Avenue, Suite 310, San Diego, CA, 92103, USA; 2 Department of Family and Preventive Medicine, University of California, San Diego, 3900 Fifth Avenue, Suite 310, San Diego, CA, 92103, USA; 3 School of Population and Public Health, University of British Columbia, 2206 East Mall, Vancouver, BC, V6T 1Z3, Canada; 4 Urban Design 4 Health, Inc., P.O. Box 78361, Seattle, WA, 98178, USA; 5 Department of Movement and Sports Sciences, Ghent University, Watersportlaan 2, Ghent, 9000, Belgium; 6 Research Foundation Flanders, Egmontstraat 5, Brussels, 1000, Belgium; 7 Department of Pediatrics, University of Washington/Seattle Children’s Research Institute, PO Box 5371, M/S CW 8-6, Seattle, WA, 98145, USA; 8 Departments of Epidemiology and Nursing, University of Pennsylvania Perelman School of Medicine and School of Nursing, 801 Blockley Hall, 423 Guardian Drive, Philadelphia, PA, 19104, USA

## Abstract

**Background:**

Streetscape (microscale) features of the built environment can influence people’s perceptions of their neighborhoods’ suitability for physical activity. Many microscale audit tools have been developed, but few have published systematic scoring methods. We present the development, scoring, and reliability of the Microscale Audit of Pedestrian Streetscapes (MAPS) tool and its theoretically-based subscales.

**Methods:**

MAPS was based on prior instruments and was developed to assess details of streetscapes considered relevant for physical activity. MAPS sections (route, segments, crossings, and cul-de-sacs) were scored by two independent raters for reliability analyses. There were 290 route pairs, 516 segment pairs, 319 crossing pairs, and 53 cul-de-sac pairs in the reliability sample. Individual inter-rater item reliability analyses were computed using Kappa, intra-class correlation coefficient (ICC), and percent agreement. A conceptual framework for subscale creation was developed using theory, expert consensus, and policy relevance. Items were grouped into subscales, and subscales were analyzed for inter-rater reliability at tiered levels of aggregation.

**Results:**

There were 160 items included in the subscales (out of 201 items total). Of those included in the subscales, 80 items (50.0%) had good/excellent reliability, 41 items (25.6%) had moderate reliability, and 18 items (11.3%) had low reliability, with limited variability in the remaining 21 items (13.1%). Seventeen of the 20 route section subscales, valence (positive/negative) scores, and overall scores (85.0%) demonstrated good/excellent reliability and 3 demonstrated moderate reliability. Of the 16 segment subscales, valence scores, and overall scores, 12 (75.0%) demonstrated good/excellent reliability, three demonstrated moderate reliability, and one demonstrated poor reliability. Of the 8 crossing subscales, valence scores, and overall scores, 6 (75.0%) demonstrated good/excellent reliability, and 2 demonstrated moderate reliability. The cul-de-sac subscale demonstrated good/excellent reliability.

**Conclusions:**

MAPS items and subscales predominantly demonstrated moderate to excellent reliability. The subscales and scoring system represent a theoretically based framework for using these complex microscale data and may be applicable to other similar instruments.

## Background

The relationship between several built environment factors and physical activity has been established in multiple reviews [1-4]. Larger characteristics, often called macro-level attributes of environments (e.g., walkability, density, street connectivity, land-use mix) are well-documented correlates of walking and physical activity [5-7]. Most of the built environment and physical activity evidence is based on macro-level variables. However, macro-level factors do not reflect the entirety of people’s experiences with their environment. “Microscale” factors include details about streets, sidewalks, intersections, and design characteristics (e.g., road crossing features, presence of trees, bicycle lanes, curbs), as well as characteristics of the social environment (e.g., stray dogs, graffiti, trash) [8]. Microscale factors may also influence physical activity [5,9,10] but have not been studied as extensively as macro-level factors. The study of microscale factors allows for a more fine-grained examination of the environmental features that enable or inhibit physical activity and may be more cost effectively and easily modified than macro characteristics.

Microscale factors are typically assessed using direct observations [5]. Many research-based microscale observation or audit tools for streetscapes (environment features in and along streets) have been developed in the past decade [5,11], and more recently, virtual audit tools using online resources like Google Streetview have been tested (maps.google.com) [12,13]. There is much heterogeneity of content across the microscale audit tools, although reviews have found common themes: building type, streets and traffic, sidewalks, bicycle facilities, public spaces/amenities, architecture/building characteristics, parking/driveways, maintenance, and safety [5,10]. As summarized by Brownson et al. [5], several microscale audit tools have been systematically developed and have demonstrated good inter-observer agreement. The current study presents methods building on these previous approaches and provides a systematic scoring system for summarizing the data, so results can be more easily interpreted.

Interpretable and policy relevant summary scores are needed to provide evidence that could be used for decisions about making the built environment more supportive of physical activity. Several groups have developed conceptual models of microscale attributes, some of which have been used as the basis for scoring systems [14-20]. These models and scoring systems are at varying stages of development, including conceptual model only [16], scoring system created but inter-rater reliability not tested or not reported [14,15,18-20], and scoring system created but based on a statistical rather than conceptual model [17,21]. These frameworks are all steps toward systematic scoring of microscale tools, but there is a need for a comprehensive approach that includes a conceptually-driven model and scoring procedures, along with evaluation of subscales’ reliability.

The present paper describes the development and evaluation of summary scores of a comprehensive microscale audit tool adapted from previous measures. We present a systematic approach to developing a scoring system, data reduction methods, and psychometric analysis for subscales. The overall goal was to create summary scores that can be used to assess detailed attributes of the built environment relevant to physical activity for use in research, city planning, and community advocacy that may promote activity-supportive environmental changes. To evaluate inter-rater reliability of subscales, the present paper combined data from three U.S. regions with varying levels of urbanity and walkability.

## Methods

### Study designs and areas

Objective microscale environmental data were collected as part of three studies examining the relation of neighborhood design to physical activity, nutrition behaviors, and weight status in children, teenagers, and older adults. These studies were conducted in urban and suburban neighborhoods in Seattle/King County, WA (children, teens, and seniors), San Diego, CA (children), and five counties in the Baltimore, MD-Washington, DC region (teens and seniors) (Table 1). As neighborhoods were selected to vary on walkability defined by macro-environment features, recreation environment, and median income, this study included a wide range of neighborhood built environment and sociodemographic characteristics [22-24] (Table 1). All studies used a GIS-derived macro-level walkability index to select neighborhoods high versus low on: net residential density, intersection density, retail floor area ratio, and mixed land use [22,25]. The data used are not publicly available. The three parent studies (NIK, TEAN, SNQLS) were approved by the San Diego State University Internal Review Board for research with human subjects.

**Table 1:** Study characteristics and designs: Neighborhood Impact on Kids (NIK) Study, Teen Environment and Neighborhood (TEAN) Study, and Senior Neighborhood Quality of Life Study (SNQLS)

**Study**	**Ages of participants**	**Regions**	**Design (quadrants sampled)**	**Eligible destinations**	**Total sample size: All routes**	**Reliability pair sample size: # routes (# segments, # crossings, # cul-de-sacs)**	**Years of MAPS data collection**
NIK	6-11 and a parent	San Diego County, CA and Seattle/King County, WA	Activity Environment^a^X Nutrition Environment^b^	Cluster of ≥3 destinations (commercial locations, parks or schools)	San Diego County: 365	San Diego County: 76 (233, 117, 16)	San Diego County: 2009
					Seattle/King County: 393	Seattle/King County: 0 (0, 0, 0)	Seattle/King County: 2009-2010
TEAN	12-16 and a parent	Seattle/King County, WA and Baltimore, MD-DC	Walkability^c^XIncome^d^	Cluster of ≥3 commercial locations, a park, or a school	Seattle/King County: 427	Seattle/King County: 72 (167, 67, 31)	Seattle/King County: 2010
					Maryland-DC: 470	Maryland-DC: 106 (42, 100, 6)	Maryland-DC: 2009-2010
SNQLS	65-97	Seattle/King County, WA	Walkability^c^XIncome^d^	Cluster of ≥3 destinations (commercial locations, parks, or school)	Seattle/King County: 462	Seattle/King County: 36 (74, 35, 0)	Seattle/King County: 2009

^a^ Defined by block group walkability and park access.

^b^ Defined by the presence or absence of grocery stores and fast food restaurants.

^c^ Walkability index defined by GIS-derived residential density, intersection density, retail floor area ratio, and mixed use.

^d^ Based on 2000 census data for block group median household income.

### Microscale Audit of Pedestrian Streetscapes (MAPS) tool development

The MAPS tool was adapted from previous tools, primarily the Analytic Audit Tool [15], as modified by the Healthy Aging Network [26], and further modified by present investigators (Additional file 1: Appendix A). Specific items thought to be relevant for seniors or youth were added to the tool for all groups of participants (e.g., sidewalk cross-slope). A cul-de-sac section was added for the youth studies because of their potential use as play areas. The MAPS tool can be found online at http://sallis.ucsd.edu.

There were four sections of the tool: overall route, street segments (defined as the area between crossings), crossings, and cul-de-sacs, as described in Table 2. Route-level variables summarized characteristics for the whole route, for variables that were likely general throughout the route (e.g., speed limit, aesthetics) or infrequent (e.g., transit stops). Segment-level variables were collected on every segment on the route. Street crossing variables were measured at every intersection or crossing on the route. Cul-de-sac variables were collected only when one or more cul-de-sacs were present within 400 feet of the participant’s home.

**Table 2:** MAPS section descriptions

**Microscale section**	**Description**
Route	•Approximately ¼ mile from a participant’s home toward a predetermined destination.
	•Included components of land use and destinations, streetscape, aesthetics and social environment
	•Consisted of varying numbers of segments and crossings within the ¼ mile.
Segment	•A section of a street between two crossings.
	•If street name changed, a new segment started.
	•There were up to 8 segments per route.
Crossing	•A crossing occurred when the rater went through an intersection, whether a pedestrian crossing existed or not.
	•There were up to five crossings per route.
Cul-de-sac	•A cul-de-sac or dead-end street had to be within 400 feet of a participant’s home.
	•The cul-de-sac was usually (but not always) the dead-end part of the participant’s street.
	•There were up to two cul-de-sacs per route.

The route section included items related to land use and destinations, transit stops, street amenities, traffic calming, hardscape and softscape aesthetics, and the social environment. The segments section assessed sidewalks, street buffers, sidewalk slope, bicycle facilities, shortcuts, visibility from buildings (“eyes on the street”), building aesthetics, trees, setbacks, and building height. The crossings section assessed crosswalks, slopes, width of crossings, crossing signals, and pedestrian protection (e.g., curb extensions, protected refuge islands). The cul-de-sacs section assessed the potential recreational environment within a cul-de-sac and included items about the size and condition of the surface area, slope, surveillance from surrounding homes, and amenities (e.g., basketball hoops).

### Route selection

Data were collected along a ¼ mile route (n = 2117 routes) starting at a study participant’s home and walking toward the nearest pre-determined destination, although not necessarily reaching a destination (Table 2). The ¼ mile designation was a major change from previous instruments and was chosen to standardize the method and limit observation time, but this approach may not be suitable for all study purposes. In each study, eligible destinations included a cluster of shops or services, a park, or a school. The shortest route from a participant’s home to the nearest eligible destination was identified using Network Analyst (ArcGIS version 9.3, ESRI, Redlands, CA, 2009). The ¼ mile endpoint was determined using Google Maps (maps.google.com). In the TEAN study in King County/WA and Baltimore-DC, data were also collected along the commercial center nearest the participant’s home. These commercial centers were identified using land use data available in GIS databases created for each study. For each route, a map was created to guide raters to the specified starting point (participant’s home or commercial center), the route to be walked, and the endpoint. The routes followed the road network (i.e., alleys and informal paths were not considered eligible routes). If the destination was reached before ¼ mile, raters continued toward the next identified destination and ended ratings there. If the destination was not reached in ¼ mile, the route ended at the conclusion of the segment that included the ¼ mile endpoint (see manual online: http://sallis.ucsd.edu). Each residential route consisted of 1 to 8 segments, 1 to 8 intersections/crossings, and up to 2 cul-de-sacs (if applicable), depending on block size and street design within the ¼ mile. Each commercial route consisted of one segment and the two intersections on either end of the segment.

### Procedures for data collection

#### Training and data collection procedure

MAPS data were collected in 2009–10. A research staff manager was responsible for training, route planning, and quality control for each data collection site cohort. Multiple raters at each study site were trained extensively to use the MAPS tool over a 3-day training and certification period (see training manual online at http://sallis.ucsd.edu). To be certified to rate independently, raters had to complete at least four route assessments with inter-rater reliability ≥95% agreement.

Raters began MAPS auditing at a participant’s residence and walked along the designated route on same side of the street. In inclement weather, raters drove 0.2% (San Diego) to 5% (Seattle) of the routes. The items on the route section were collected across the entire ¼ mile. When the rater crossed the street, either at a designated intersection with or without a pedestrian crossing, or due to an obstruction in the walkway, a new crossing section was completed, along with a new segment section. When a street changed names, a new segment section was started. Cul-de-sacs or street dead-ends that were within 400 feet of a participant’s home were rated using the cul-de-sac section (child and teen studies only).

All routes in the reliability sample were completed by a combination of two people drawn from the pool of certified raters. The two raters independently coded 13.7% of participants’ ¼ mile routes for reliability purposes. The second ratings were completed within 1-week of the first, typically on different days. Each rater traveled the route independently and completed assessments without consulting with the other. The final reliability analysis sample included 290 route pairs, 516 segment pairs, 319 crossing pairs, and 53 cul-de-sac pairs.

The majority of the tools were completed using paper and pencil, though some were completed using a tablet PC. Residential route sections were completed in 28.5 minutes on average (range = 5-120 minutes) and commercial route sections were completed in 18.5 minutes on average (range = 2-75 minutes). Raters and coordinators reviewed each tool for missing and discrepant items. If more than 5% of items were missing, raters returned to the route and completed the missing items. Google Earth was used to fill in missing items when re-rating was not possible (approximately 1% of cases), and to verify outliers and slope measurements.

### Conceptual approaches to the MAPS scoring system

**Figure 1:**
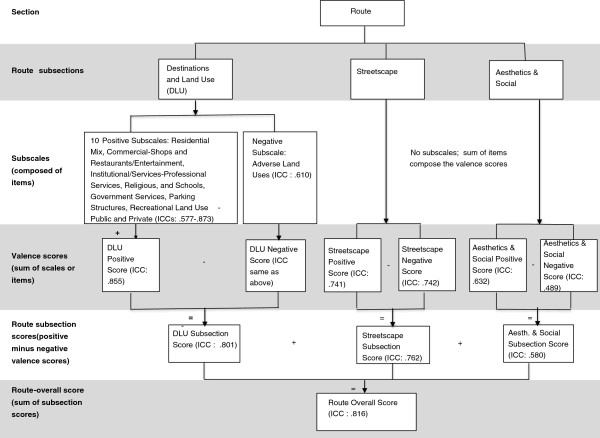
**MAPS scoring structure and summary of inter-rater reliability: Route section (one survey).** Notes: Intraclass Correlation Coefficient (ICC). The “ + ” and “-” are used to indicate arithmetic functions. For example, subscale scores are added to create valence scores. Negative valence scores are subtracted from positive valence scores to create overall section scores.

**Figure 2:**
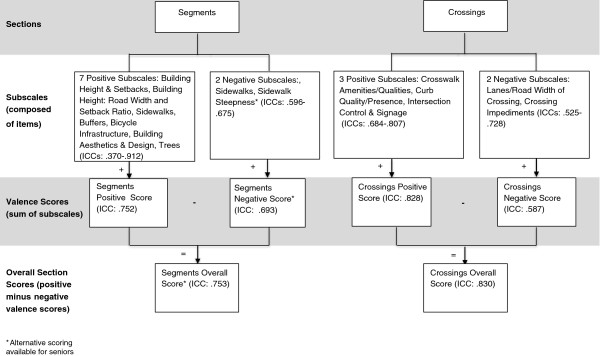
**MAPS scoring structure and summary of inter-rater reliability: Segments and Crossings sections (multiple surveys per route).** Notes: Intraclass Correlation Coefficient (ICC). The “ + ” and “-” are used to indicate arithmetic functions. For example, subscale scores are added to create valence scores. Negative valence scores are subtracted from positive valence scores to create overall section scores.

An a priori conceptual framework for scoring was created based on relevant literature and expert opinion [10,17-20,27,28]. The theoretical framework was guided by a combination of factors thought to influence people’s perceptions of their physical activity environments: safety, aesthetics, destinations, arterial or thoroughfare roads, land use, recreational facilities, transportation environment, and social environment/physical incivilities [17,19,20,27]. Combining these theoretical elements into the framework of the MAPS tool’s sections (route, crossings, segments, cul-de-sacs) resulted in a tiered classification system of items into subscales at multiple levels of aggregation. Decisions about creation of scores and scales were made based on expert consensus (members of the study team), theoretical determinations, and to create maximally policy relevant subscales. For example, different agencies could be expected to control policies related to the three route subscales: land use, streetscape, and aesthetics. Figures 1 and 2 illustrate the hierarchy of scores from lowest to highest level of aggregation. The route section had three subsections (destinations and land use, streetscape, and aesthetics and social), and subscales were computed as a precursor of higher levels of scale aggregation. All sections and subsections had positive and negative valence scores, based on expected effect on physical activity, except cul-de-sacs, which only had a single (positive) score. Negative valence scores were subtracted from positives to create subsection scores for routes. Finally, omnibus scores were created for each of the three main sections (route, crossings, segments).

### Item-level scoring and data analysis

Items from each of the three main MAPS sections were sorted a priori into subscales based on group consensus (see http://sallis.ucsd.edu for item placement into subscales and results tables for sample items in each subscale). Scoring conventions were developed and when possible, were used to simplify scoring. Most items in MAPS were coded dichotomously (no/yes) and scored as 0/1. Frequency items (0, 1, 2+) were scored as 0, 1, 2 and continuous and descriptive items were dichotomized or trichotomized based on their distributions, theoretical relevance, and in compatibility with other scale items’ scoring. In several instances, related items needed to be combined into single variables to be meaningful components of their respective subscales. For example, sidewalk and walking path presence were collected as two separate items, but for interpretation they were combined to create one variable for the subscale. In such cases, the new variable was computed and then coded for scoring (e.g., di- or trichotomized) consistently with theoretically related items and to match with the other scale items’ scoring. The slope items in the segment section were scored separately for seniors compared to the other age groups. The rationale was that seniors may be more sensitive to higher slopes than younger people, so their activity may be negatively affected at lower grade slopes.

The Kappa statistic was used to assess the inter-rater reliability of dichotomous variables, accounting for the effects of chance agreement [29]. Kappa values were judged using a modified version of Landis and Koch’s [30] adjectival classification system: “good to excellent” (≥0.60), “moderate” (0.41-0.60), or “fair to poor” (≤0.40) [31]. Items with poor agreement (Kappa or ICC <0.40) were dropped from scales unless expert opinion (of those on the study team) determined their necessity/relevance. For example, some item reliabilities were poor due to low frequency. Items excluded from scale creation were kept in the MAPS tool.

Continuous or ordinal items were assessed for inter-rater reliability using one-way random effects single-measure intraclass correlation coefficients (ICC). Despite the lack of consensus in interpreting ICC values, the scale of ICCs is similar to that of Kappa, and so the classification system for Kappa values described above was also used to evaluate ICC values [32]. Because low variability can affect Kappa and ICC values, the percent agreement was also calculated for all variables [19,31,33]. Percent agreement was classified using the following criteria (modified from [19,31]: “good to excellent” (≥75%), “moderate” (60-74%), and “fair to poor” (<60%)). Items were considered acceptable if one or both measures of reliability were moderate or higher. All individual item statistics, including those from dropped items, and syntax for creating item transformations and subscales can be found at: http://sallis.ucsd.edu.

All data were analyzed using SPSS version 17.0 (SPSS, Inc., Chicago, IL). For each item (original and modified), frequency, range, mean, standard deviation, and inter-rater reliability were calculated.

### Subscale creation and analysis

Scales were created and analyzed based on the reliability samples from all studies combined. After the remaining (not dropped) items were transformed as necessary, the subscale scores were computed by summing the items’ scores. The subscales were then sorted by expected positive or negative effects on physical activity, to create the valence scores (see Figures 1 and 2). For instance, the sum of the positive destinations and land uses was thought to be positively associated with physical activity, and the presence of social disorder was thought to be negatively associated. All of the positive subscales were summed to create the positive valence score, and likewise for the negative subscales into the negative valence score. Finally, an overall section score (positive–negative valence scores) was calculated for each of the three main sections. Total MAPS positive, negative, and overall scores can be calculated by summing the three main sections’ positive, negative, and overall summary scores.

For the subscales and the positive/negative/overall scores, frequency, range, mean, standard deviation, and inter-rater reliability (ICC and % agreement) were also assessed using the acceptability criteria described above. Subscale reliability scores were considered to be acceptable if ICC values were moderate or higher. In some cases, several iterations of subscales were run, with step-wise exclusion of low reliability items considered important conceptual indicators. If inclusion of low reliability but relevant items did not reduce the subscale’s reliability below acceptable standards, the items in question were included.

## Results

The number of segments and intersections, and their characteristics, varied across neighborhoods (Table 3). Individual item reliabilities are available online (http://sallis.ucsd.edu/). Forty-one items out of 201 (20.4%) were dropped from subscale creation due to low reliability, low frequency, text responses, or lack of consensus on theoretical relevance or expected direction of influence. Dropped route items included senior/paratransit stops, drainage ditches, historical/cultural features, other obstructions to walking, presence of anyone walking, and litter items. Dropped segment items included how much of the segment is at the steepest slope, signs discouraging skateboard use, and dead-end streets (separate from cul-de-sacs). Dropped crossings items included crosswalk timing, bike lanes through a crossing, unanticipated mid-segment crossings, and lack of street lamps. The cul-de-sac items dropped due to lack of expert consensus about their directionality of hypothesized influences on physical activity were percentage of cul-de-sac or dead-end paved, number of driveways entering the cul-de-sac or dead-end area, having an island in the cul-de-sac, and access through the cul-de-sac to another street or area.

**Table 3:** Average census block group-level descriptive statistics of reliability sample areas (mean, SD)

**Study site**	**Median household income ($)**	**Median age (years)**	**Percent of residents with college degree or higher (%)**	**Percent residents non-Hispanic White (%)**	**Net Residential Density**^**a**^	**Intersection Density**^**b**^	**Retail Floor Area Ratio**^**c**^	**Mixed Use**^**d**^
San Diego County, CA	47,293 (14,715)	35.0 (6.8)	21.0 (16.1)	62.2 (24.1)	3.98 (3.35)	46.64 (20.59)	0.22 (0.15)	0.20 (0.16)
Seattle/King County, WA	52,267 (19,704)	36.1 (5.2)	38.3 (18.9)	70.6 (20.8)	8.56 (7.25)	61.12 (21.27)	0.27 (0.15)	0.31 (0.19)
Baltimore, MD-DC	49,874 (19,991)	38.0 (6.2)	34.7 (24.5)	62.6 (28.6)	9.66 (8.54)	49.34 (24.79)	0.33 (0.21)	0.33 (0.19)
Overall	49,590 (17,767)	36.0 (6.2)	30.0 (20.6)	65.2 (24.2)	6.80 (6.67)	52.29 (22.60)	0.26 (0.17)	0.27 (0.19)

^a^ Residential units/acres of residential land.

^b^ Number of intersections per square kilometer.

^c^ Ratio of total building floor area to total parcel area.

^d^ Entropy measure of building floor area for mixed use (uses include 5 categories: civic and educational, entertainment, commercial, multi- and single-family, office).

### Subscale reliability

#### Route

Table 4 provides route subscale, valence score, subsection score, and overall route score components, descriptive statistics, sample items, and reliability statistics. The item reliability statistics presented are for the original tool items, not the recoded or transformed variables. Included in the subscales were 11 (12.2%) route items with low reliability (defined by ICC or Kappa), 18 (20.0%) with moderate reliability, and 44 (48.9%) with good/excellent reliability; 17 items (18.9%) were so rare that no Kappa or ICC could be calculated, in addition to the 14 excluded items. The low reliability and rare variables were retained in the analyses due to their theoretical importance. Of the included items for which Kappa or ICC could be calculated, 84.9% had moderate to excellent reliability.

**Table 4:** Route subscale characteristics: all studies combined (n = 290 route reliability pairs)

**Subscale**	**# items (range of scores)**	Sample items* and overall subscale description	**Mean (SD)**	**ICC, % agreement**	**Range of item ICCs or Kappas**
**Land Use and Destinations Subscales**					
Positive Subscales					
Residential Mix (weighted residential density)	4 (0–3)	Single family homes, apartments/condominiums, apartments above street retail	1.34 (.64)	.577, 80.0%	.292 (retirement/senior living facilities) - .776
Commercial-Shops	10 (0–11)	Food-related land uses, retail and service-oriented land uses and shopping centers	1.37 (2.25)	.873, 74.2%	.407 -.842
Commercial-Restaurants/Entertainment	4 (0–6)	Food-related uses (fast food, sit-down, café), entertainment	.799 (1.31)	.842, 81.3%	.765-.796
Institutional/Services-Professional Services	3 (0–6)	Bank/credit union, health-related professional, other services	1.26 (1.54)	.849, 75.3%	.743-.808
Institutional/Services-Religious, Schools (each a single item)	1 each (0–2 each)	Government or community land use: Place of worship; school	N/A	Religious: .712 Schools: .722	Same as subscales
Government Services	4 (0–4)	Health or social services, library/museums, post office, senior center	.135 (.436)	.652, 91.4%	.279 (senior center) -.798
Parking Structures (positive influence on PA)	2 (0–2)	No parking facilities present, parallel/angled on-street parking	1.61 (.79)	.736, 80.0%	-.011 (no parking) -.689
Recreational Land Use-Public Recreation Facilities	4(0–3)	Community garden, public indoor, public outdoor pay, public park	.179 (.466)	.717, 91.6%	.497-.679
Recreational Land Use-Private Recreation Facilities	2 (0–2)	Private indoor, private outdoor	.097 (.34)	.696, 95.5%	.659-.704
DLU Commercial (an interim subscale, may be used independently, but not included in overall scores)	3 subscales (0–21)	Sum of shops, restaurant/entertainment, and services subscales. Subscale created to reflect most common pedestrian destinations. Not included in overall positive subscale.	3.39 (4.40)	.889, 50.3%	See above
DLU Overall Positive Subscale	10 subscales (2–24)	Sum of subscales: residential mix, shops, restaurants/entertainment, services, government services, religious, schools, positive parking, public recreation, and private recreation.	7.08 (4.51)	.855, 43.8%	See above
Negative Subscale					
Adverse Land Uses: Industrial, Abandoned Lot/Building, Surface Parking Lot or Garage **ALSO IS DLU Overall Negative	6 (0–7)	Warehouse/factory/industrial, abandoned building, large parking facilities	1.17 (1.24)	.610, 96.3%	-.029 (abandoned building)-.659
Overall Subscale					
DLU Overall Subscale Score	2 subscales (0–21)	DLU Overall Positive subscale – Adverse Land Uses subscale	5.91 (4.71)	.801, 37.7%	See above
**Streetscape Subscales**					
Positive Elements Subscale	18 (0–10)	Transit stops, posted speed limit, pedestrian signage, street amenities (e.g., working telephone, trash bins)	3.70 (2.16)	.741, 49.8%	.395 (presence of kiosks or info booths) - .838
Negative Elements Subscale	5 (0–4)	High speed limits, roll-over curbs, driveways	1.69 (.876)	.742, 70.1%	.433-.814
Overall Streetscape Score	2 subscales (−3 – 10)	Positive Streetscape Elements subscale– Negative Streetscape Elements subscale	2.01 (2.66)	.762, 45.7%	See above
**Aesthetics and Social Subscales**					
Positive Aesthetics and Social Subscale	5 (0–5)	Public art, landscaping maintenance	2.08 (1.09)	.632, 48.7%	.391 (signage for commercial destinations or parks) -.689
Negative Aesthetics and Social Subscale	14 (0–8)	Graffiti, physical disorder, broken windows	1.91 (1.81)	.514, 36.6%	.088 (social disorder (dichot: none vs. any) -.665
Overall Aesthetics and Social Score	2 subscales (−8 – 5)	Positive Aesthetics and Social -Negative Aesthetics and Social Subscales	0.18 (2.52)	.580, 29.5%	See above
**Total Route Score**	3 overall subscales (−2 – 33)	Sum of 3 overall scores	7.94 (8.18)	.816, 17.4%	See above

*All item reliabilities can be found at: http://sallis.ucsd.edu.

For the destinations and land use route section, there were ten positive subscales and one negative subscale (Table 4; Figure 1). The positive destinations and land use subscales included residential mix (weighted residential density scores), shops, restaurants/entertainment, professional services, religious, schools (each of the latter two a single item), government services, positive parking structures, public recreational land use, and private recreation facilities. Nine out of ten (90%) of the positive destinations and land use subscales had good to excellent inter-rater reliability, with ICC (or Kappa for the two items) values ranging from .652 (government services) to .873 (shops). The one subscale with moderate agreement was housing mix (ICC: .577). The overall positive destinations and land use valence score was a sum of all ten positive subscales and had good/excellent reliability (ICC: .855). The negative destinations and land use valence score consisted only of the adverse land uses and demonstrated good/excellent reliability (ICC: .610; Table 4). The destinations and land use subsection score (positive valence score - negative valence score) had good/excellent reliability (ICC: .801).

Due to the low number of items and relative homogeneity of item content, the route streetscape items were aggregated into a positive or negative valence score, and the streetscape subsection score (positive–negative valence scores; Figure 1). The positive streetscape items that were thought to influence walking included items such as pedestrian signage and street amenities (e.g., drinking fountains, trash cans; Table 4). The negative items that were thought to deter walking included items such as high speed limits, multiple driveways, and non-barrier curbs. All of the streetscape valence and overall scores had good/excellent inter-rater reliability: positive (ICC: .741), negative (ICC: .742), and overall (ICC: .762).

The positive route aesthetics and social subscale had the same structure as for streetscape: positive and negative valence scores, and an aesthetics and social subsection score (positive–negative valence scores; Figure 1). The positive items thought to influence walking included availability of public art, landscaping, and general aesthetic maintenance and the positive valence score had good/excellent reliability (ICC: .632). The negative items thought to negatively influence walking included physical disorder, broken windows, and graffiti. The negative aesthetics and social valence score had moderate reliability (ICC: .514) as did the overall aesthetics and social subsection score (ICC: .580).

The overall route score was calculated from the sum of the three route subsections scores (destinations and land use, streetscape, and aesthetics and social). The route overall score had good/excellent reliability (ICC: .816). For the route valence and subsection scores, three out of three for destinations and land use had good/excellent reliability, for streetscape, three out of three had good/excellent reliability, and for aesthetics and social, one out of three had good/excellent reliability, with two having moderate reliability. In sum, of the route valence, subsection, and overall scores, seven out of nine (77.8%) had good/excellent reliability, and the remaining two (22.2%) had moderate reliability.

#### Segments

Of the items included in the subscales, there were zero segment items (0%) with low reliability, 14 items (46.7%) with moderate reliability, and 16 items (53.3%) with good/excellent reliability; there were no items with incalculable ICC or Kappa, and there were 5 items excluded from subscales. There were seven positive segment subscales and two negative segment subscales, with separate child/teen and senior coding for the negative sidewalk steepness subscale (Table 5; Figure 2). The positive subscales included building height and setbacks, the building height: road width and setback ratio, sidewalk positive qualities, buffers (between street and sidewalk), bicycle infrastructure, building aesthetics and design, and trees. Six out of seven (85.7%) of the positive subscales had good/excellent reliability, with ICCs ranging from .614 (building height: road width and setback ratio) to .921 (buffers). The building height and setbacks subscale was the exception, demonstrating poor reliability (ICC: .370). The positive segment valence score (sum of the seven positive subscales) had good/excellent reliability (ICC: .752). The negative subscales included sidewalk negative qualities and sidewalk steepness (separately scored for children/teens and seniors). One of the negative subscales demonstrated good/excellent reliability (ICC: .675 for sidewalk negative qualities), and the other had moderate reliability (ICC: .596 for sidewalk steepness for children/teens). The negative valence score for children/teens (sum of the two negative subscales including sidewalk steepness for children/teens) had good/excellent reliability (ICC: .693). The negative valence score for seniors (sum of the two negative subscales including sidewalk steepness for seniors) also had good/excellent reliability (ICC: .689). The overall segment section score for children/teens (positive -negative valence scores for children/teens) had good/excellent reliability (ICC: .753). The overall segment section score for seniors (positive -negative valence scores for seniors) also had good/excellent reliability (ICC: .758). In sum, all five of the segment valence and overall section scores had good/excellent reliability.

**Table 5:** Segment subscale characteristics: all studies combined (n = 516 segment reliability pairs with complete sidewalk data)

**Subscale**	**# items (range)**	Sample items* and overall subscale descriptions	**Mean (SD)**	**ICC, % agreement**	**Range of item ICC or Kappa**
Positive Subscales					
Building Height and Setbacks	3 (0–4)	Smallest and largest setbacks and building height	1.31 (.644)	.370, 69.0%	.522-.764
Building Height: Road Width and Setback Ratio	3 (0–3)	Smallest and largest setbacks, building height, and road width	.103 (.457)	.614, 97.1%	n/a
Sidewalk Positive Qualities	3 (2–3)	Sidewalk presence and width	2.23 (.419)	.555, 84.6%	.489-1.00
Buffers	2 (0–2)	Buffer presence and width	.826 (.941)	.912, 93.0%	.882-.919
Bicycle Infrastructure	2 (0–3)	Marked bicycle lane, signage	.200 (.706)	.855, 95.4%	.676-.791
Building Aesthetics and Design	4 (0–7)	Street-level windows, building colors and materials	3.85 (1.81)	.705, 38.0%	.549 - .629
Trees	3 (0–5)	Number and spacing of trees, percent of sidewalk shaded	2.15 (2.08)	.744, 51.2%	.540-.737
Informal Path (single item)	1 (0–1)	Is there an informal path (shortcut) which connects to something else?	n/a	.554 (K), 91.1%	n/a
Overall Positive	7 subscales plus 1 item (3–22)	Sum of subscales: building height and setbacks, sidewalk positive qualities, buffers, bike infrastructure, building aesthetics and design, trees, plus item: cul-de-sac connectivity	10.78 (3.29)	.752, 25.4%	See above
Negative Subscales					
Sidewalk Negative Qualities	5 (0–4)	Trip hazards, obstructions in the sidewalk	1.09 (1.02)	.675, 55.5%	.494-.796
Sidewalk Steepness (children/teens)	3 (0–5)	Slope, cross-slope (steeper slope acceptable for children)	1.09 (1.01)	.596, 60.1%	.503- .775
Sidewalk Steepness (seniors)	3 (0–7)	Slope, cross-slope (less steep slope acceptable for seniors)	2.18 (1.64)	.633, 42.4%	.502- .746
Overall Negative Subscale (Child/Teen)	2 subscales (0–7)	Sum of subscales: Sidewalk negative qualities, sidewalk steepness (children/teens), building height: road width and setback ratio, negative street design/width	2.18 (1.63)	.693, 42.4%	See above
Overall Negative Subscale (Senior)	2 subscales (0–9)	Sum of subscales: Sidewalk negative qualities, sidewalk steepness (seniors), building height: road width and setback ratio, negative street design/width	3.27 (2.01)	.689, 33.4%	See above
Overall Subscales					
Overall Segments Score (Child/Teen)	2 (−1 -19)	Overall Positive – Overall Negative subscales (child/teen)	8.58 (3.43)	.753, 24.0%	See above
Overall Segments Score (Senior)	2 (−2 -19)	Overall Positive – Overall Negative subscales (senior)	7.49 (3.66)	.758, 22.8%	See above

*All item reliabilities can be found at: http://sallis.ucsd.edu.

#### Crossings

There were 7 items (31.4%) with low reliability, 8 items (17.2%) with moderate reliability, and 12 items (42.9%) with good/excellent reliability; 1 item (8.5%) was too rare to calculate ICC or Kappa, and 8 items were excluded from subscales. There were three positive crossings subscales: crosswalk amenities/qualities, curb quality/presence, and intersection control and signage (Table 6; Figure 2). All three of these subscales had good/excellent reliability, with ICCs ranging from .684 (curb quality/presence) to .807 (crosswalk amenities/qualities). The positive crossing characteristics valence score had good/excellent reliability (ICC: .828). There were two negative crossings subscales: lanes/road width of crossing and crossing impediments. Of the two negative subscales, the crossing impediments subscale had good/excellent reliability (ICC: .728), and the lanes/road width of crossing subscale had moderate reliability (ICC: .525). The negative crossing characteristics valence score (sum of the two negative subscales) had moderate reliability based on ICC (.587). The overall crossings section score (positive–negative valence scores) had good/excellent reliability (ICC: .830). In sum, two out of three of the valence and overall crossing subscales had good/excellent reliability and one had moderate reliability.

**Table 6:** Crossing subscale characteristics: all studies combined (n = 319 crossing reliability pairs)

**Subscale**	**# items (range)**	Sample items* and overall subscale description	**Mean (SD)**	**ICC, % Agreement**	**Range of item ICC or Kappa**
Positive Subscales					
Crosswalk Amenities/Qualities	9 (0–4)	Crosswalk characteristics (e.g., marked crosswalk, high visibility markings)	.987 (1.08)	.807, 70.2%	-.012 (curb extensions) - .816
Curb Quality/Presence	2 (0–2)	Pre- and post-crossing curb lining up with crossing	1.33 (.91)	.684, 82.4%	.648-.651
Intersection Control and Signage	10 (0–7)	Stop signs, pedestrian walk signals	1.28 (1.31)	.752, 71.6%	.327 (traffic circle) - .811
Overall Positive Crossing Characteristics Subscale	3 subscales (0–12)	Sum of subscales: crosswalk amenities/qualities, curb quality/presence, intersection control and signage	3.61 (2.53)	.828, 54.2%	See above
Negative Subscales					
Lanes/Road Width of Crossing	1 (0–2)	Distance of crossing leg (# lanes wide, trichotomized)	.764 (.581)	.525, 72.9%	n/a
Crossing Impediments	7 (0–4)	No curb ramp, gutters in crossing, faded/worn crosswalk markings	1.14 (1.17)	.728, 72.9%	.188 (poor visibility at corners) - .893
Overall Negative Crossing Characteristics Subscale	2 subscales (0–5)	Sum of subscales: Lanes/Road Width of Crossing, Crossing Impediments	1.53 (1.48)	.587, 61.2%	See above
Overall Subscale					
Overall Crossings Score	2 subscales (−4-8)	Sum of subscales: Overall Positive Crossing Characteristics-Overall Negative Crossing Characteristics	2.05 (2.27)	.830, 42.9%	See above

*All item reliabilities can be found at: http://sallis.ucsd.edu.

#### Cul-de-sacs

There were 53 cul-de-sac reliability pairs. Sample items included questions about the size of the cul-de-sac, smoothness of the pavement, and whether parking was allowed. Twelve items were included in the subscale, and fourteen were excluded. Of the included items, none had low reliability, one had moderate reliability, 8 had good/excellent reliability (highest ICC: .809), and 3 items (25.0%) were too rare to calculate Kappa or ICC. The twelve items with consensus on directionality (for impact on physical activity) were summed to create one overall positive subscale with a scores ranging from 3 to 9. The subscale had a mean of 6.5 (SD: 1.59), and it demonstrated good/excellent reliability (ICC: .847).

## Discussion

A systematic coding scheme was developed for the MAPS streetscape observation tool that summarized 160 items into 26 specific subscales, 10 valence scores, and one final overall score for each of the MAPS sections: route, crossings, segments, and cul-de-sacs. A majority of items (75.6%) and all but one of the subscales (96.1%) had moderate or good/excellent reliability. The most common items with lower agreement were those assessing slope, subjective qualities (e.g., the social environment/disorder), and those with complex response options (e.g., percentage of sidewalk shaded by trees, number of façade types/colors). When necessary, reconceptualization, recoding, and editing were carried out for under-performing items and scale scores. This scoring and scale development approach allows examination of microscale attributes at varying and tiered levels of specificity and complexity. The guiding assumption was that the aggregate environmental impact is likely to be most influential on physical activity. It is possible that critical positive or negative single factors can override an aggregate score, and the present scoring system allows examination of detailed items, specific conceptual categories, or aggregate scores. Thus, all coding and scoring decisions were made considering expected effects on physical activity, but the scales may be relevant for other outcomes, such as social interactions, social capital, perceptions of environments, and neighborhood satisfaction as well.

Numerous microscale tools have been developed [5], reflecting a recognition that the details of streetscapes are likely to affect experience and behavior beyond macro-level variables (e.g., street connectivity) that are studied much more often. However, the lack of well-developed and accessible scoring systems, along with the expense of data collection, seems to have inhibited the use of microscale tools, because there are few published reports using microscale data [9,27,34,35]. The complexity of the measures also appears to have been a deterrent to developing scoring systems because of differing response scales, item formats, and conceptual domains. Many of the scoring systems that have been developed have not been tested for inter-rater reliability or reliability has not been reported in the literature [14-16,19,21,36]. Thus, the present scoring system represents an advance, and the principles used may be applicable to other microscale tools.

Though all published microscale measures assess a wide range of attributes, MAPS may be more comprehensive because it assesses and provides scores for routes, crossings, segments, and cul-de-sacs. Present results are expected to be generalizable, at least to metropolitan areas in the United States, because data came from three geographic regions (Baltimore, MD/Washington, DC, San Diego, CA, and Seattle, WA) and included neighborhoods representing a wide range of urban design and income. Though MAPS has not been used in rural areas, it could be applicable to such settings as well, although further development of micro-scale evaluation in such areas is needed.

The innovative approach of evaluating routes beginning with each study participant's home was adopted for two major reasons. First, the routes were highly personalized and designed to reflect the environment each participant would likely encounter while walking or bicycling to a nearby destination. Second, the route approach was efficient and controlled the time cost of assessing each person’s local environment. Selecting routes was appropriate for the parent studies because the participants’ locations were known. Other microscale tools were designed to audit a sample of street segments, and then create summary scores that can be related to individual behavior [9,19,20,33]. However, there are major questions about how many segments or what percent of neighborhood streets need to be observed to represent an adequate sample [5]. Observed streets may or may not be representative of the environment experienced by a given participant. The larger the areas over which environmental characteristics are averaged, the less valid they are likely to be for each participant. Thus, observation of individual routes has important procedural advantages for some types of studies and is likely to produce more valid estimates of effect sizes in relation to physical activity and other outcomes.

Another advantage of the scoring system outlined in the present paper is its generalizability. The principles used to score MAPS may be applicable to other audit tools. The principles included the combination of conceptual- and policy-driven scoring systems, items scored as dichotomies or trichotomies, hypothesis-driven classification into positive or negative subscales for expected effects on physical activity, and the inclusion of a hierarchy of scores from construct-specific to aggregated. The likely utility of this approach includes the benefit that specific construct measures may be useful for guiding city planning and transportation decisions. It also may help residents understand the strengths and weaknesses of their micro-scale (street and pedestrian) environments, including assessing local disparities in environmental quality. The overall scores and aggregated scores may be better suited for explaining outcomes because cumulative effects across numerous attributes are more likely than individual items to influence behavioral outcomes and neighborhood perceptions [37].

### Limitations

The MAPS scoring and reliability process included several limitations. The decision-making processes for item retention and scale creation was driven by theory [28,38,39], research team discussion, statistical criteria, policy-relevance, and expert consensus. While these strategies included accepted methods for such decision-making, there were ambiguous cases and situations. The originally proposed conceptually-driven process had to be modified throughout the course of data reduction. While we present one method of combining items and subscales, subtracting negatives from positives may not reflect the complexity of various microscale elements. Ultimately, scales were created with the aforementioned blend of theory, practicality, and expert consensus in hopes that this method may prove useful to wider audiences and researchers. The present scoring system may not be optimal, and other approaches to combining items and examining patterns of attributes should be explored.

A limitation of the design is that the selected route may not be a direction usually taken by the participant, or a participant may take multiple routes to the destination(s) of interest. However, the route selection procedure was designed to identify a likely route in the direction of the nearest destination. The most important environmental attributes (e.g., a high speed roadway) may have been just beyond the ¼ -mile designated route. This issue can be better addressed in forthcoming validity analyses.

Quantifying the diversity of built environment variables is inherently complicated, and rarely does one answer fit all situations. Given that the focus of the present scoring process was on influences relevant to physical activity and the desire to link variables with planning and transportation policies, some of the decisions may have created scales that do not apply to all potential future uses. For example, present scoring may not be generalizable if scoring neighborhoods for relevance to social capital or in very different contexts such as Asian or African cities. Further, the three regions sampled herein likely do not represent the entire range of environments that MAPS may be used in the future. During analysis, many of the dropped items were determined to contain ambiguous wording or concepts. Future investigators can revise or exclude items in a modified MAPS but should carefully describe changes and additions, including validation of any changes.

Typical methods of scale development include factor analysis and assessment of internal consistency (i.e., Cronbach’s alpha), but they were not used in the present study. These approaches are appropriate for psychological measures but may not be for environmental assessment. Features that are conceptually related (e.g. street lights and speed limit for safety) may not co-exist, so internal consistency is not a sufficient index of scale quality. The present goal was to create conceptually coherent and policy-relevant scales. By identifying clusters of environmental attributes under the jurisdictions of planning, transportation, and public works departments, results can be more relevant for establishing accountability for making environmental improvements that would be expected to promote physical activity.

### Next steps

The next analytic step is to conduct validity analyses that will relate these scale scores to total physical activity and specific walking outcomes (e.g., walking to school, for leisure, for transport) in multiple age groups. Another next step would be to develop an electronic-based interface for integrated data collection and scoring (e.g., iPad app). Following validation, these scales can be used for research and practice purposes. As tools like MAPS are used in different environments and countries, and by investigators interested in different outcomes, the tool itself is likely to be modified. It is difficult to imagine how a standard version could be developed that could be used for community intervention and policy advocacy purposes and be comparable across locations. As one step toward more frequent use of streetscape audits, validity analyses could result in a shorter form containing the most valid items and scales.

## Conclusions

This paper presets a theoretically based policy relevant scoring system for the MAPS environmental audit tool. The modest number of interpretable scores makes the tool more useable by researchers, policy makers, and practitioners. The MAPS tool and scoring system demonstrated strong reliability and can be recommended for wider use.

## Competing interests

No authors report competing interests.

## Authors’ contributions

RM played lead roles in writing the manuscript, conducting data analysis, and creating the conceptually based scoring system. KC participated in study design and coordination, data management, analysis and interpretation, and helped to draft the manuscript. CG coordinated data collection, participated in data management, and helped to draft the manuscript. JS, LF, and BS combined and modified existing micro-scale tools into MAPS, participated in study design and data collection and management, and edited the manuscript. JC helped with route selection, study design and data collection. TC helped design the MAPS tool and data collection protocol, helped conceptualize scale scoring and statistical analysis, and edited the manuscript. DVD helped with conducting the reliability analyses and edited the manuscript. LD helped with the study coordination and edited the manuscript. JK edited the manuscript. KG edited the manuscript and assisted with study design. All authors read and approved the final manuscript.

## Pre-publication history

The pre-publication history for this paper can be accessed here:

http://www.biomedcentral.com/1471-2458/13/403/prepub

## Supplementary Material

Additional file 1: Appendix A MAPS Tool Development. Click here for file
