# Supplementary material for: Development, scoring, and reliability of the Microscale Audit of Pedestrian Streetscapes (MAPS)
Source: BMC Public Health. 2013 Apr 27;13:403. doi: 10.1186/1471-2458-13-403 (PMC3728214; doi:10.1186/1471-2458-13-403)
Supplement: Additional file 1: Appendix A — MAPS Tool Development. [file 1471-2458-13-403-S1.pdf]

MAPS Tool Development

Land Use/Destinations (Route section)

MAPS items	Source	Modification for MAPS?
1-4	PRC-HAN [1, 2]	No
5	Created by investigative team	N/A
6a-ad	PRC-HAN	Yes, response format and types of land uses: added ‘Specialty Food Store’, ‘School’, ‘Casino’
7	Created by investigative team	N/A

Streetscape (Route section)

MAPS items	Source	Modification for MAPS?
1	PRC-HAN	Yes, removed non-relevant response options; count for each
2	PRC-HAN	Yes, changed ‘none’ to ‘neither’
2	Created by investigative team	N/A
3	PRC-HAN	Yes, changed to select the highest speed limit to get route level estimate
4	PRC-HAN	Yes, removed ‘cul-de-sac’ as an option; count for each
5-6	PRC-HAN	No
7	PRC-HAN	Yes, added ‘kiosks or information booths’
8	Created by investigative team	N/A

Aesthetics and Social (Route section)

**MAPS items**	**Source**	**Modification for MAPS?**
1-2	PRC-HAN	Yes, split item to assess separately hardscape and softscape features
3	PRC-HAN	Yes, removed ‘artistic’ because asked about in above items
4-5	PRC-HAN	Yes, modified wording of question and response format accordingly
6a-h	PRC-HAN	No
6i-j	Created by investigative team	N/A
7-8	PRC-HAN	No
9	PRC-HAN	Yes, removed response options that were likely dependent upon time of day
10	PRC-HAN	Yes, simplification of this item to make it less complicated to rate and less temporal

Segments

MAPS items	Source	Modification for MAPS?
1	PRC-HAN	Yes, only one side
2	PRC-HAN	Yes, only one side; take out ‘both’ option
3a	PRC-HAN	Yes, only one side
3b	Created by investigative team	N/A
4-9	PRC-HAN	Yes, only one side
10	Created by investigative team	N/A
11 (one-way/two-way)	Irvine-Minnesota [3]	
12	PRC-HAN	Yes, only one side; took out “in street option”
13-14	PRC-HAN	Yes, only one side
15 (bicycle signs)	PIN3 Neighborhood Audit Instrument [4]	
16 (skateboard signs)	Created by investigative team	
17	Irvine-Minnesota	
18	Irvine-Minnesota	
19	Observational Validation of Urban Design Measures for New York City [5]	
20-21	Observational Validation of Urban Design Measures for New York City	
22 (material)	Observational Validation of Urban Design Measures for New York City	
23-28	Created by investigative team	N/A

Crossings

MAPS items	Source	Modification for MAPS?
1	PRC-HAN	No
2	PRC-HAN	No
3	PRC-HAN	No
4	PRC-HAN	Yes, take out estimate of adequacy of timing to cross
5	PRC-HAN	Yes, changed response options to be consistent with crossing level and non temporal
6	Created by investigative team	N/A
7a-c	PRC-HAN	Yes, removed 'map or info kiosk' and 'large print street signs'
8a-e	PRC-HAN	Yes, combined 'stop lines' and 'additional warning' option; added 'different material than road' option
9	Created by investigative team	N/A
10	PRC-HAN	No
11	PRC-HAN	Yes, changed 'refuge islands' to 'protected refuge islands'; removed 'center median strip', 'especially wide lanes' and 'angled intersection'
12	PRC-HAN	Removed 'missing or faded street signs'

References

1. Kealey M, Kruger J, Hunter R, Ivey S, Satariano W, Bayles C, Ramirez B, Bryant L, Johnson C, Lee C, Levinger D, Mctigue K, Moni C, Moudon AV, Pluto D, Prohaska T, Sible C, Tindal S, Wilcox S, Winters K, Williams K: **Engaging Older Adults to Be More Active Where They Live: Audit Tool Development.** In *Proceedings of the 19th National Conference on Chronic Disease Prevention and Control: March 2005; Atlanta*.

2. Caughy MO, O'Campo PJ, Patterson J: **A brief observational measure for urban neighborhoods.** *Health Place* 2001, **7**:225-36. Retrieved from http://www.ncbi.nlm.nih.gov/pubmed/11439257
3. Boarnet MG, Day K, Alfonzo M, Forsyth A, Oakes M: **The Irvine-Minnesota inventory to measure built environments: reliability tests.** *Am J Prev Med* 2006, **30:**153-159. doi:10.1016/j.amepre.2005.09.018
4. Evenson KR, Sotres-Alvarez D, Herring AH, Messer L, Laraia BA, Rodríguez DA: **Assessing urban and rural neighborhood characteristics using audit and GIS data: Derivation and reliability of constructs.** *Int J Behav Nutr Phys Act* 2009, **6:**44. doi:10.1186/1479-5868-6-44
5. Ewing R, Clemente O, Handy S, Brownson RC, Winston E: **Identifying and measuring urban design qualities related to walkability.** Prepared for the Active Living Research Program of the Robert Wood Johnson Foundation, July 2005.
